# Long non-coding RNA GAS5 promotes cisplatin-chemosensitivity of osteosarcoma cells via microRNA-26b-5p/TP53INP1 axis

**DOI:** 10.1186/s13018-023-04387-z

**Published:** 2023-11-22

**Authors:** Guowei Li, Xue Yan

**Affiliations:** 1 Department of Spine Surgery, The First Affiliated Hospital of Jinzhou Medical University, Jinzhou, 121000 Liaoning China; 2 Respiration Medicine, The First Affiliated Hospital of Jinzhou Medical University, No. 2, Section 5, Renmin Street, Guta District, Jinzhou, 121000 Liaoning China

**Keywords:** Osteosarcoma, GAS5, miR-26b-5p, TP53INP1, Resistance, DDP

## Abstract

Osteosarcoma is a common malignant bone tumor. Cisplatin (DDP) achieves a high response rate in osteosarcoma. Here we aim to study the dysregulation of long non-coding RNA the growth arrest-specific transcript 5 (GAS5), and its roles in DDP-resistance of osteosarcoma. The expression of mRNA and microRNA in osteosarcoma tissues and osteosarcoma cell lines were detected by quantitative reverse-transcription polymerase chain reaction, and protein expression levels were measured by western blotting assay. Cell Counting Kit-8 and 5-Ethynyl-2′-deoxyuridine were used to measure cell proliferation. Flow cytometer assay was used to evaluate cell apoptosis. The interactions between miR-26b-5p and GAS5 or tumor protein p53-induced nuclear protein 1 (TP53INP1) were verified by dual luciferase reporter along with biotin RNA pull-down assays. GAS5 was identified to be significantly lowly expressed in osteosarcoma samples especially in cisplatin-resistant (DDP-resistant) tissues. GAS5 was also downregulated in DDP-resistant cells. Over-expressed GAS5 prominently increased the sensitivity of osteosarcoma cells to DDP in vitro. Furthermore, over-expression of GAS5 suppressed cell proliferation and facilitated apoptosis of DDP-resistant cells. Mechanistically, GAS5 sponged miR-26b-5p, over-expression of which reversed the effects of GAS5 on cell proliferation and apoptosis of DDP-resistant cells. In addition, miR-26b-5p targeted TP53INP1. TP53INP1 abrogated the functions of miR-26b-5p on cell proliferation and apoptosis in DDP-resistant cells. Taken together, GAS5 enhanced the sensitivity of osteosarcoma cells to DDP via GAS5/miR-26b-5p/TP53INP1 axis. Therefore, GAS5 may be a potential indicator for the management of osteosarcoma.

## Introduction

Osteosarcoma is the most common primary malignant tumor of the bone and primarily arises in children and adolescents. After the combined treatment of neoadjuvant chemotherapy as well as surgical resection, the 5-year survival rate of patients with osteosarcoma increased from 10–20% to 60–70%, but the prognosis of osteosarcoma patients resistant to chemotherapy is poor, and are prone to recurrence and metastasis [1, 2]. Tumor metastasis is closely related to chemotherapy resistance. However, increasing the dose of first-line therapy or applying second-line chemotherapy to patients who do not respond well to chemotherapy has very limited efficacy [3, 4]. Therefore, it is necessary to explore the potential mechanism of osteosarcoma chemotherapy resistance.

Long non-coding RNA (lncRNA) is a sub-type of RNA elements without protein-coding capability [5, 6]. LncRNAs participate in regulating cell resistance in a variety of ways, including increasing drug metabolism, enhancing drug efflux, changing cell cycle, affecting abnormal apoptosis and epithelial-mesenchymal transformation (EMT) [7]. Li et al. [8] discovered that up-regulated HOTTIP decreased the sensitivity of osteosarcoma cells to DDP by activating the Wnt/β-catenin pathway. Furthermore, elevated LINC00161 increased DDP-induced apoptosis and reversed the DDP-resistant phenotype of osteosarcoma cells by upregulating IFIT2 [9]. Therefore, we speculated that lncRNAs may serve as regulatory factors on drug resistance of osteosarcoma to DDP. Recent evidence suggests that lncRNA growth arrest-specific transcript 5 (GAS5) is an antioncogene in diverse tumors, and GAS5 enhanced the sensitivity of tumor cells to chemotherapy or radiotherapy [10, 11]. As report goes, GAS5 suppresses proliferation and invasion of osteosarcoma cells by sponging different microRNAs (miRNAs) [12, 13]. However, the chemotherapy-related resistance potentials of GAS5 in osteosarcoma has not been reported.

The role and molecular mechanism of GAS5 in DDP-resistance of osteosarcoma were investigated in vivo and in vitro. Briefly, GAS5 expression levels in osteosarcoma tissues and cell lines were detected. The effects of GAS5 over-expression on proliferation and apoptosis of osteosarcoma DDP-resistant cell lines were analyzed. The miRNA that have binding sites with GAS5 was predicted. This may provide a new treatment method for DDP-resistance of osteosarcoma.

## Materials and methods

### Bioinformatic analyses

Online database Starbase 3.0 (http://starbase.sysu.edu.cn/), Targetscan 7.1 (http://www.targetscan.org/vert_71/), and miRDB (http://www.mirdb.org/) were used to predict interactions between mRNA and miRNA [14–16]. RNAfold web server (http://rna.tbi.univie.ac.at//cgi-bin/RNAWebSuite/RNAfold.cgi) was applied to analyze the secondary structures of GAS5 [17]. Enrichment analysis of target genes were performed by FunRich software (http://www.funrich.org/) [18].

### Patients

Tumor tissues along with corresponding matched adjacent normal tissues of 60 patients with osteosarcoma from January 2008 to December 2012 in the First Affiliated Hospital of Jinzhou Medical University were collected in this study. Of the 60 subjects, 30 patients underwent three cycles of DDP (120 mg/m^2^) and were assessed for progression of disease (PD) according to Response Evaluation Criteria in Solid Tumors version 1.1 (RECIST 1.1) [19], and another 30 patients did not receive any chemotherapy prior to enrollment. All participants have signed informed consent. Our study has been approved by the Ethics Committee of the First Affiliated Hospital of Jinzhou Medical University according to Declaration of Helsinki.

### Cell culture

Human immortalized osteoblasts hFOB1.19 and four osteosarcoma cell lines (U2OS, HOS, Saos2, and MG63) were cultured in DMEM/F12 added with 10% inactivated fetal bovine serum (Giboc, USA), which was replaced every 2–3 days. Cells at 80–90% conflunce were digested and used in the following experiment.

### Establishment of DDP-resistant cells

Logarithmic growth Saos2 or MG63 cells were inoculated into the cell plates and cultured in 0.1 μg/mL DDP solution (Nanjing Pharmaceutical Company, China) for 2 days. Then the culture medium was replaced with DDP-free medium. When the cell density reached over 85%, the subculture was performed. The above operation was repeated three times to increase the DDP concentration to 0.2 μg/mL. After about 17 weeks, the resistant cells were cultured in 2 μg/mL DDP solution (MG63/DDP and Saos2/DDP). In some experiments, sensitive and DDP-resistant cells were treated with DDP at the concentration of 2 μg/mL.

### Lentiviral transfection and RNA interference

Logarithmic growth MG63/DDP cells were digested and cultured in 6-well plates overnight. shRNA negative control (shRNA nc), shRNA GAS5, LV negative control (vector) and LV over/GAS5 (GAS5) lentivirus vectors (all purchased from Genepharma Company, Shanghai, China) were added to infect the cells. In order to obtain a stable GAS5 over-expressed or down-regulated cell lines, the lentivirus infected cells were selected by incubation with 2 μg/mL of puromycin. Then expression of GAS5 of MG63/DDP cells stably infected was examined by quantitative reverse-transcription polymerase chain reaction (qRT-PCR).

miR-26b-5p mimic, miR-26b-5p inhibitor, and their negative controls (purchased from Genepharma Company, Shanghai, China) were transfected into cells of each group by using Lipofectamine 3000 (Invitrogen, USA) under the guidance of the manufacturer’s instructions.

### qRT-PCR

Total RNA of clinical samples and osteosarcoma cells was extracted via an RNA extraction kit (Beijing Tiengen Biochemical Technology Co., LTD, China). cDNA was obtained using a reverse transcription kit (Genecopoeia, USA). PCR reaction was conducted according to SYBR Premix Ex Taq kit instructions (Takara, Japan), pre-denaturation at 95 °C for 30 s, then 40 cycles of denaturation at 95 °C for 5 s, annealing at 60 °C for 30 s. GAPDH was used as housekeeping control for GAS5 and tumor protein p53-induced nuclear protein 1 (TP53INP1). U6 was used as housekeeping control for miR-26b-5p. The data have been analyzed using 2^−ΔΔCt^ relative expression method. Thrice experiments were repeated.

### Cell proliferation

Cell viability was evaluated by Cell Counting Kit-8 (CCK-8) method. Cells were inoculated in 96-well plates at a concentration of 2 × 10^4^ cells per mL, and 100 μL cell suspension was inoculated in 96-well plates. After cultured in the incubator for 2, 24, 48 and 72 h, 10 μL CCK-8 solution (Thermo Fisher Scientific, USA) was added, and cultured for 2 h, respectively. The optical density (OD) at 450 nm wavelength was detected on a microplate reader.

Cell proliferation was measured by 5-Ethynyl-2′-deoxyuridine (EdU) method. Logarithmically grown osteosarcoma cells were inoculated into 96-well plates with 1 × 10^4^ cells per well and cultured in a cell incubator for 24 h. 50 μL EdU was added to each well at 37 °C for 2 h. After that, 100 μL 4% neutral paraformaldehyde buffer was added to fix cells for 0.5 h. Then cells were permeated with 0.5% Tritons-X and incubated with 100 μL Hoechst 33,342 for 0.5 h. The EdU positive cells was observed under a fluorescence microscope (Eclipse E600; Nikon, Japan).

### Flow cytometry

1 × 10^6^ cells were inoculated in a cell culture dish with a diameter of 10 cm and lysed with 0.25% trypsin. After centrifugation, the supernatant was removed and the cells were diluted with PBS. Annexin V-FITC and PI were added to the cells. The results were detected by flow cytometry (Bioscience, USA).

### Fluorescence in situ hybridization (FISH)

Alexa Fluor 555-labeled GAS5 probes were designed and synthesized by RiboBio (Guangzhou, China). The probe signals were determined with a FISH Kit (RiboBio, Guangzhou, China) under the guidance of the instruction book, and pictures were captured by a fluorescence microscope (Eclipse E600; Nikon, Japan).

### Western blotting assay

The apoptosis-related protein expressions of cleaved-caspase-3 (ab2302, 1:100), Bax (ab182735, 1:1000), and Bcl-2 (ab32124, 1:1000) were detected by western blotting assay. After conventional culture for 24 h, the cells were added with protein lysate. The supernatant was collected and the protein density was measured with a protein quantification kit. 30 μg protein was separated using SDS-PAGE; after separation, the protein was transferred to polyvinylidene fluoride membrane, which was then blocked with 5% fresh defatinated milk powder at room temperature for 1 h. The primary antibodies (Abcam, USA) were added and incubated successively (overnight at 4 °C, 12–16 h), and HRP labeled secondary antibody (at room temperature for 1 h) was added to avoid light for color development (31460, Invitrogen, USA). GAPDH (ab8245,1: 1000, Abcam, USA) was used as internal reference. The gray value of each band has been analyzed by gel image analysis software (Alpha Innotech, USA). Thrice experiments were repeated.

### Dual luciferase reporter assay

The miR-26b-5p overexpressed plasmids and its control plasmids were co-transfected into MG63/DDP cells with GAS5 or TP53INP1 3’UTR wild type (PGL3-GAS5 3’UTR-WT or PGL3-PTTG1 3’UTR-WT) reporting vector and mutant reporting vector (PGL3-GAS5 3’UTR-MUT or PGL3-PTTG1 3’UTR-MUT), respectively. 48 h later, the medium supernatant was absorbed, PLB lysate (K801-200; BioVision Tech, USA) was added to each well and lysed for 15 min. The lysate was collected and the luciferase activity was evaluated by a luciferase activity kit.

### Biotin RNA pull-down assay

Biotin-bound transcripts of miR-26b-5p (biotin-miR-26b-5p) and negative control (biotin-nc) synthesized by Integrated Biotech Solutions (Shanghai, China) were, respectively, transfected into MG63/DDP cells. Then MG63/DDP cells were lysed for 48 h after transfection. Then 50 μl beads-containing solution (Life Technologies, USA) was added in cell lysate. After sufficient mixed, TRIzol Reagent (Life Technologies, USA) was applied to withdraw RNA from the beads. The results were detected by qRT-PCR.

### Statistical analysis

Data analysis was performed by Graphpad Prism (Version 8.0, GraphPad software, USA). Each independent experiment was conducted triplicate. Data were expressed as mean ± standard deviation. Student t test has been used for comparison between the two groups and one-way ANOVA was used for comparison between the multiple groups. *P* < 0.05 was deemed to be statistically significant.

## Results

### GAS5 was reduced in DDP-resistant osteosarcoma cells

**Fig. 1:**
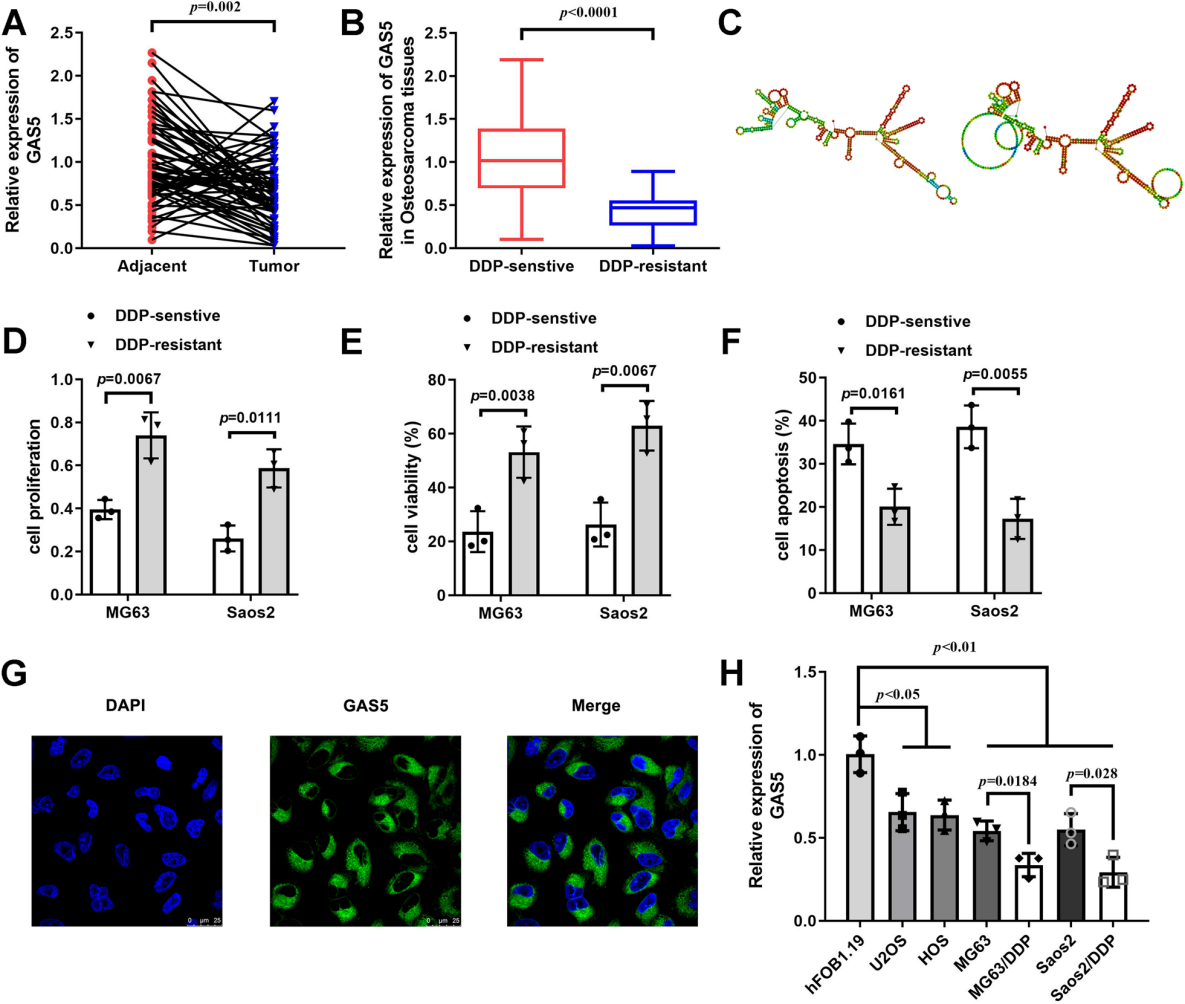
GAS5 was reduced in DDP-resistant osteosarcoma cells. **A** qRT-PCR analyses of GAS5 expression in osteosarcoma tissues and adjacent controls. **B** qRT-PCR analyses of GAS5 levels in osteosarcoma tissues from DDP-resistant and DDP-sensitive patients. **C** The secondary structures of GAS5 analyzed by RNAfold web server. **D**–**F**. Cellular functions measured by CCK-8, EdU staining and flow cytometry assays. **G** GAS5 was mainly localized in the cytoplasm of osteosarcoma cells analyzed by FISH. **H** mRNA level of GAS5 in osteoblast and osteosarcoma cell lines including two DDP-resistant cells

GAS5 was downregulated in osteosarcoma tissues compared with that in adjacent normal group (Fig. 1A), among which the levels of GAS5 were further suppressed in osteosarcoma tissues obtained from DDP-resistant patients (Fig. 1B). GAS5 expression in osteosarcoma tissues was used as the basis for clinicopathological analysis, and there was no statistical significance between clinical parameters and GAS5 expression (Table 1). As shown in Fig. 1C, the secondary structures of GAS5 analyzed by RNAfold web server contain multiple stem rings and multiple branched internal rings. What’s more, DDP treatment motivated cell proliferation (Fig. 1E, F) and alleviated cell apoptosis (Fig. 1F) of MG63 and Saos2 cells. Considering that miRNAs are transported from the nucleus to the cytoplasm [20], we examined the localization of GAS5 to determine whether it can bind to miRNAs. FISH analysis revealed that GAS5 was primarily localized in the cytoplasm in MG63/DDP cells (Fig. 1G). Meanwhile, expression level of GAS5 was dramatically suppressed in osteosarcoma cell lines in contrast with osteoblasts hFOB1.19, and was further suppressed in DDP-resistant osteosarcoma cells compared with DDP-sensitive ones (Fig. 1H).

**Table 1:** Clinical parameter of the enrolled subjects

Clinical parameter	Cases (n = 60)	Expression levels		*P* value
		GAS5^hign^	GAS5^low^	
Gender				0.4348
Male	26	15	11	
Female	34	15	19	
Age (years old)				0.7961
	28	13	15	
	32	17	15	
Tumor size (cm)				01799
< 5 cm	22	14	8	
≥ 5 cm	38	16	22	
TNM stage				0.0169
I	24	17	7	
II + III	36	13	23	
Distant metastasis				0.6094
Yes	31	17	14	
No	29	18	11	

### Over-expression of GAS5 suppressed DDP-resistance in osteosarcoma cells

**Fig. 2:**
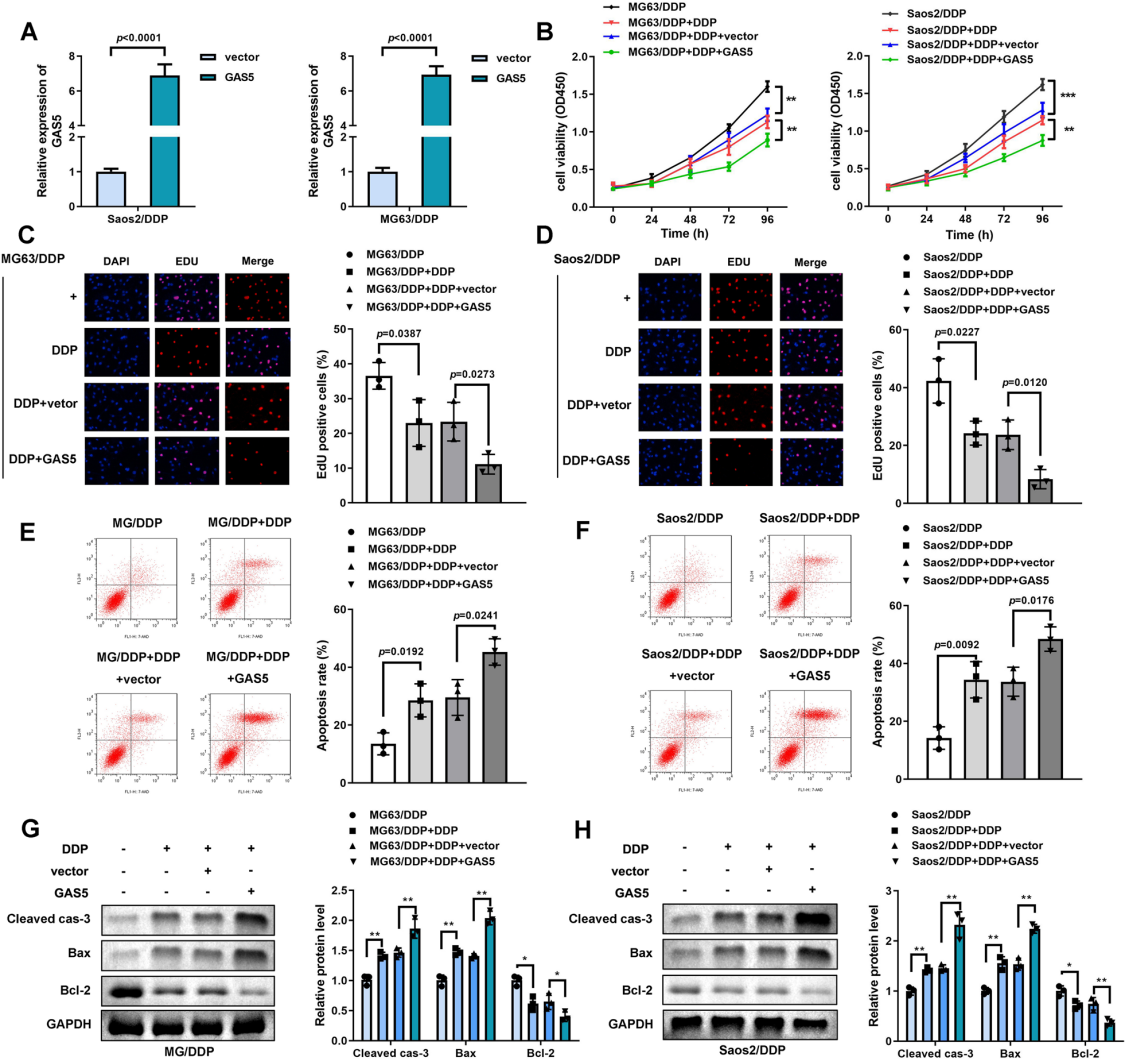
Suppression of GAS5 inhibited cell viability and facilitated apoptosis of DDP-resistant osteosarcoma cells. **A** qRT-PCR analyses of GAS5 expression in MG63/DDP and Saos2/DDP cells after lentiviral transfection. **B** Cell viability evaluated by CCK-8 assay in MG63/DDP and Saos2/DDP cells treated with DDP and transfected with GAS5, **P < 0.01; ***P < 0.001. **C**, **D**. Cell proliferation detected by EdU assay in MG63/DDP and Saos2/DDP cells treated with DDP and transfected with GAS5. **E**, **F**. Cell apoptosis determined by flow cytometry assay in MG63/DDP and Saos2/DDP cells treated with DDP and transfected with GAS5. **G**, **H**. Western blotting assay analyses of expression levels of cleaved-caspase-3, Bax, as well as Bcl-2 in MG63/DDP and Saos2/DDP cells treated with DDP and transfected with GAS5, *P < 0.05; **P < 0.01. The concentration of DDP is 2 μg/mL

Figure 2A reveals that the expression of GAS5 was significantly induced in cells transfected with over-expressed GAS5 vectors, indicating successful transfection. DDP-induced decrease in cell proliferation and increase in apoptosis rates was further facilitated by over-expressed GAS5 in MG63/DDP and Saos2/DDP cells (Fig. 2B–F). What’s more, the protein expression of cell apoptosis related proteins were evaluated, and the results demonstrated that cleaved-caspase-3 and Bax were up-regulated while Bcl-2 was down-regulated by over-expression of GAS5 (Fig. 2G, H).

### GAS5 acted as a miR-26b-5p sponge in DDP-resistant osteosarcoma cells

**Fig. 3:**
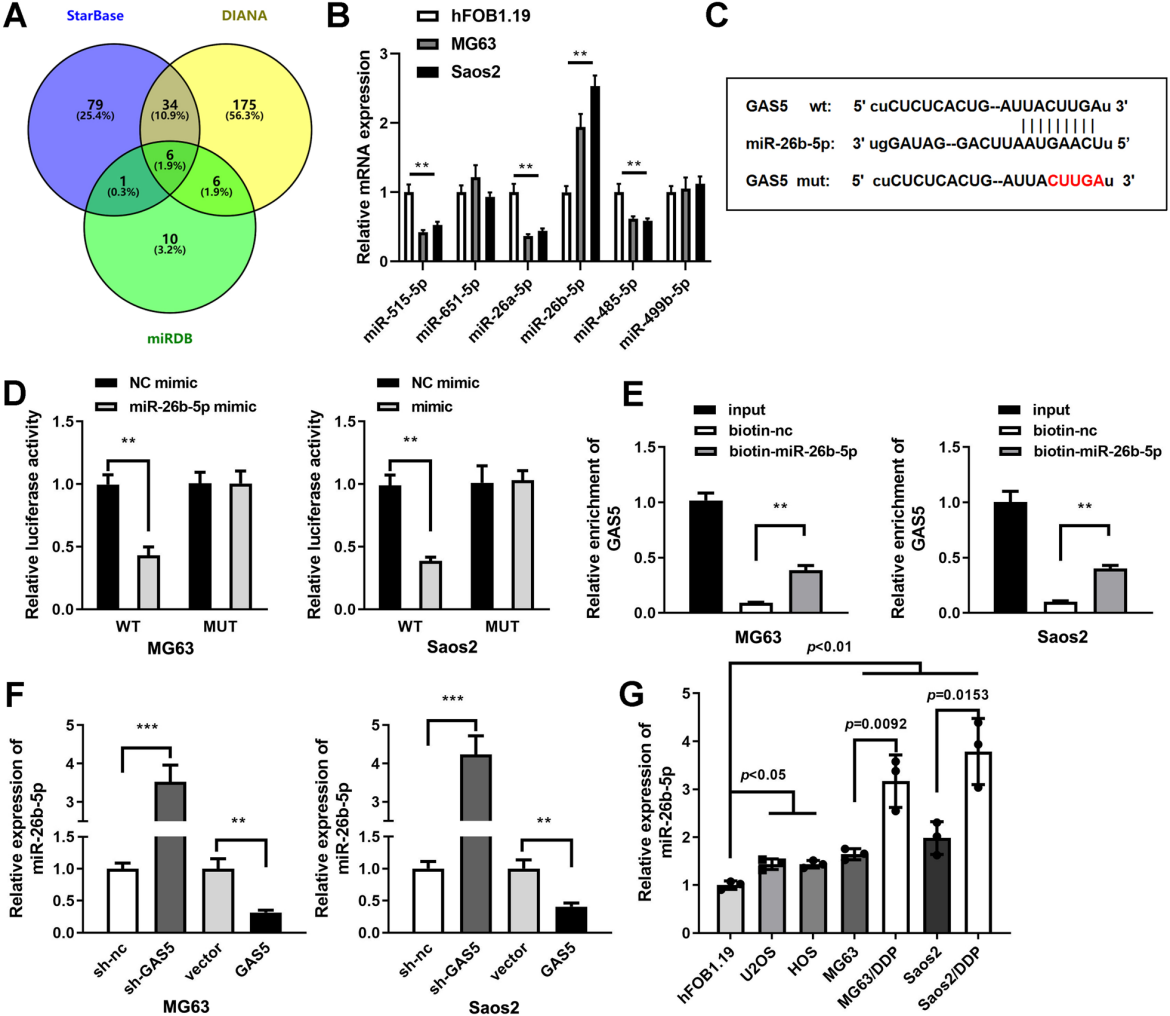
GAS5 acted as a miR-26b-5p sponge in MG63/DDP and Saos2/DDP cells. **A** Venn diagram of miRNAs that bind with GAS5 were predicted through three bioinformatic tools. **B** mRNA level of 6 miRNAs in hFOB1.19 and osteosarcoma cells, **P < 0.01. **C** Interactions between miR-26b-5p and wild-type GAS5. **D** Dual-luciferase reporter assay demonstrated that miR-26b-5p bind with wild-type GAS5 3’UTR, **P < 0.01. **E** Biotin RNA pull-down assay results demonstrated that GAS5 was gathered in miR-26b-5p biotin probe compared to negative control, **P < 0.01. **F** mRNA expression levels of miR-26b-5p after down-regulation of GAS5 or over-expression of GAS5, **P < 0.01; ***P < 0.001. G. qRT-PCR analyses of miR-26b-5p in osteoblast and osteosarcoma cell lines including two DDP-resistant cells

Six common miRNAs that can bind to GAS5 were screened out from three online databases (Fig. 3A), and then expression levels of 6 candidates were obtained at cellular level. miR-26b-5p was notable for its significantly high expression level in osteosarcoma cells (Fig. 3B). Figure 3C shows the binding sites between miR-26b-5p and 3’UTR wild-type of GAS5. Then luciferase activity was obviously repressed in cells transfected with miR-26b-5p mimic and 3’UTR wild-type of GAS5 (Fig. 3D). Biotin RNA pull-down assay results demonstrated that GAS5 was enriched in miR-26b-5p biotin probe compared with negative control (Fig. 3E). miR-26b-5p expression was negatively regulated by GAS5 (Fig. 3F). Finally, miR-26b-5p expression levels were notably up-regulated in osteosarcoma cell lines compared to osteoblasts hFOB1.19, which was more remarkable in MG63/DDP and Saoa2/DDP cells (Fig. 3G).

### Up-regulated miR-26b-5p abrogated the functions of GAS5 on cell proliferation as well as apoptosis of DDP-resistant osteosarcoma cells

**Fig. 4:**
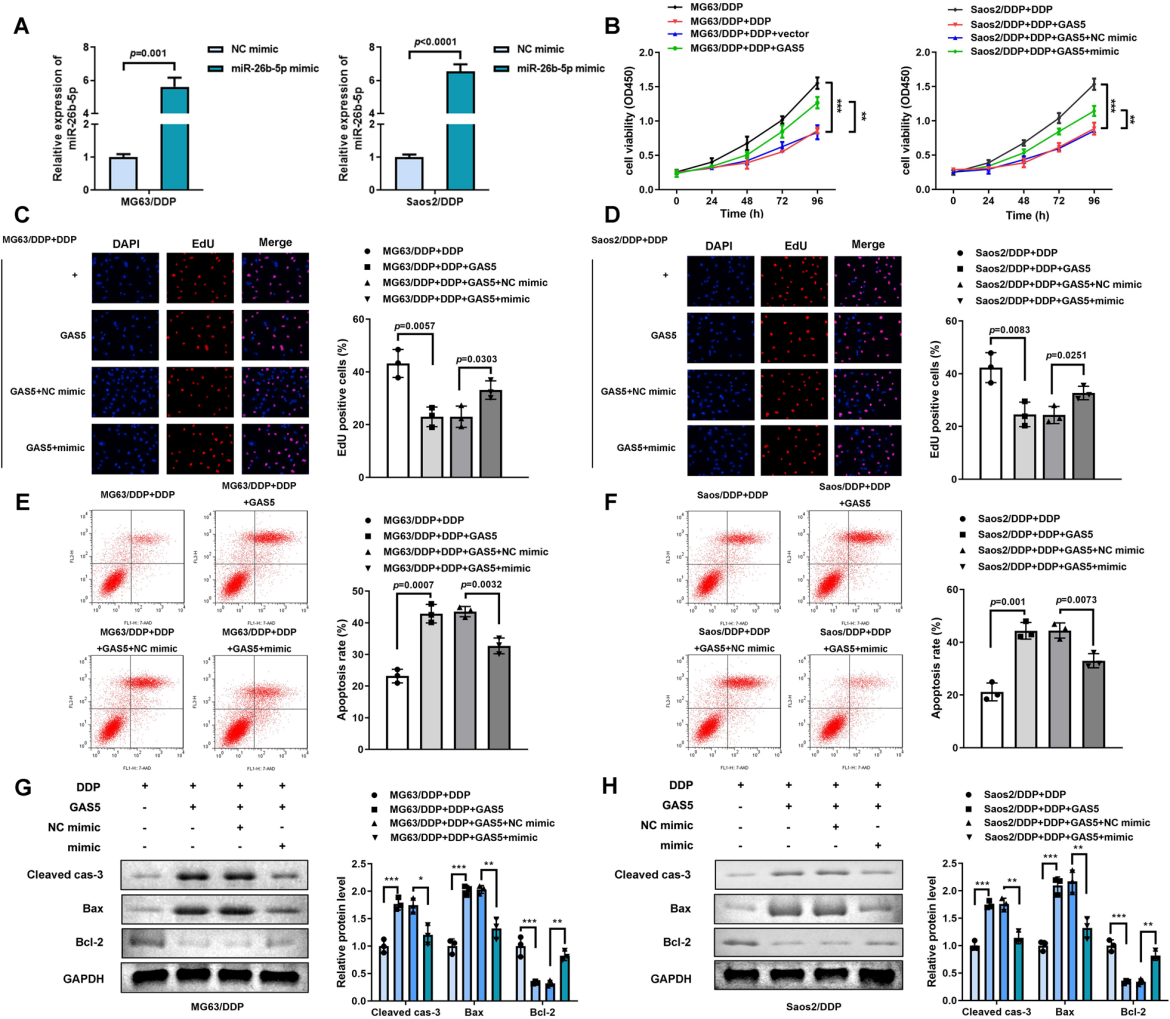
Up-regulated miR-26b-5p reversed the effects of GAS5 on cellular functions of MG63/DDP and Saos2/DDP cells. **A** miR-26b-5p expression levels of MG63/DDP and Saos2/DDP were determined by qRT-PCR after transfection. **B**. Cell viability detected by CCK-8 assay in MG63/DDP and Saos2/DDP cells treated with DDP and co-transfected with GAS5 and miR-26b-5p over-expression plasmids, **P < 0.01; ***P < 0.001. **C**, **D**. Cell proliferation detected by EdU assay in MG63/DDP and Saos2/DDP cells treated with DDP and co-transfected with GAS5 and over-expressed miR-26b-5p plasmids. **E**, **F** Cell apoptosis determined by flow cytometry assay in MG63/DDP and Saos2/DDP cells treated with DDP and co-transfected with GAS5 and up-regulated miR-26b-5p plasmids. **G**, **H** Western blotting assay analyses of expression levels of caspase-3, Bax, and Bcl-2 in MG63/DDP and Saos2/DDP cells treated with DDP and co-transfected with GAS5 and miR-26b-5p mimic, *P < 0.05; **P < 0.01; ***P < 0.001. The concentration of DDP is 2 μg/mL

The level of miR-26b-5p was obviously induced in miR-26b-5p mimic group (Fig. 4A). Elevated miR-26b-5p dramatically alleviated the effects of GAS5 and promoted cell proliferation (Fig. 4B–D) and suppressed cell apoptosis (Fig. 4E, F). Furthermore, inhibition of miR-26b-5p partly reversed the regulatory role of GAS5 on apoptosis-related protein expression of cleaved-caspase-3, Bax, and Bcl-2 (Fig. 4G, H).

### TP53INP1 was a downstream gene of miR-26b-5p

**Fig. 5:**
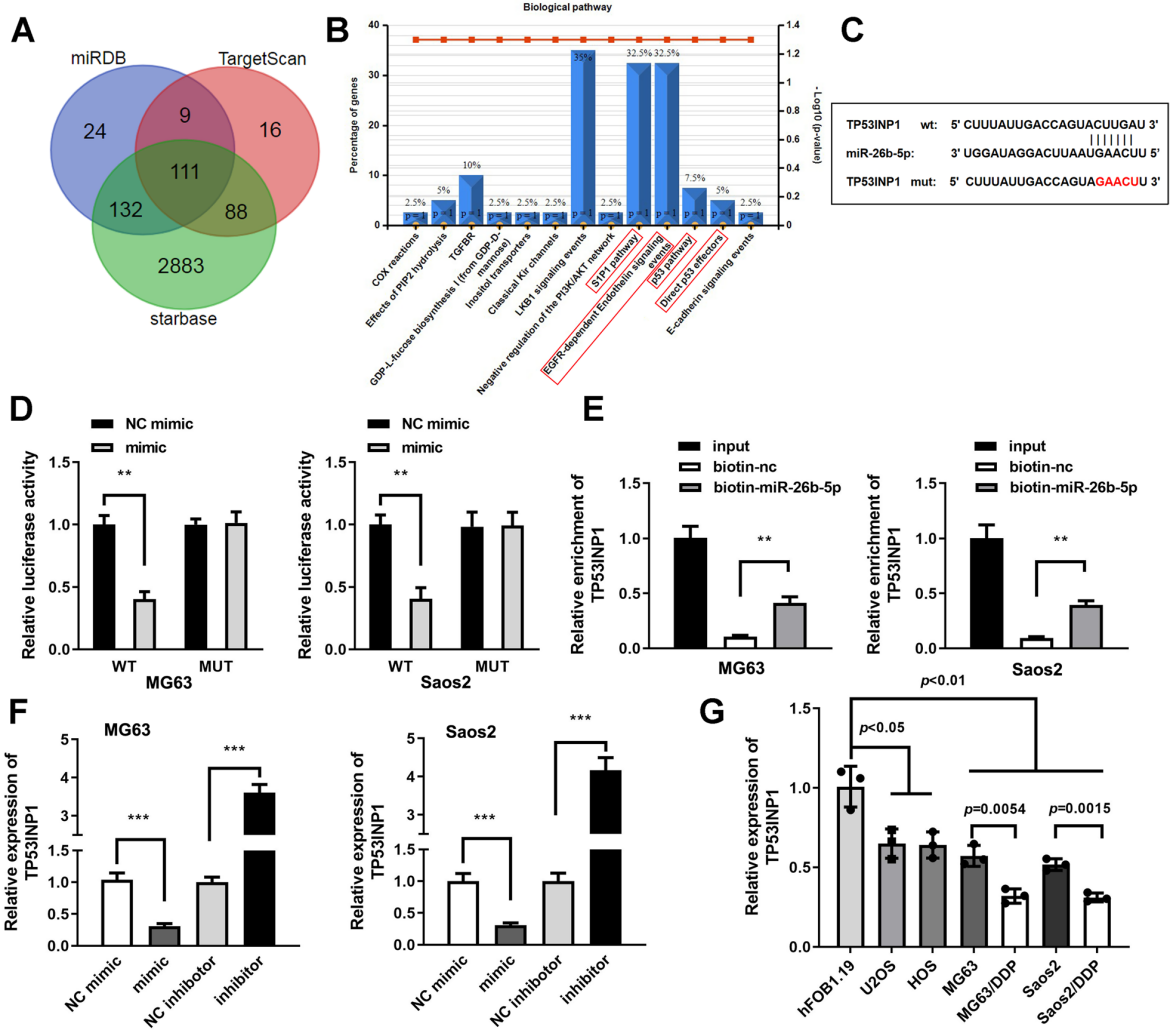
TP53INP1 was a target gene of miR-26b-5p. **A** Venn diagram of downstream genes of miR-26b-5p were predicted through three bioinformatic tools. **B** Biological pathways of 111 genes enrichment analyzed by FunRich. **C** Binding sites between miR-26b-5p and wild-type TP53INP1. **D** Dual-luciferase reporter assay demonstrated that miR-26b-5p bind with wild-type TP53INP1 3’UTR, **P < 0.01. **E** Biotin RNA pull-down assay results demonstrated that TP53INP1 was gathered in miR-26b-5p biotin probe compared with negative control, **P < 0.01. **F** mRNA expression levels of TP53INP1 after transfection, ***P < 0.001. **G** qRT-PCR analyses of TP53INP1 in osteoblast and osteosarcoma cell lines including two DDP-resistant cells

Then we selected 111 downstream genes of miR-26b-5p (Fig. 5A), and the FunRich analyses demonstrated that p53 pathway was enriched in the top ten biological pathways. Therefore, the target gene TP53INP1 which can prominently improve the activity of p53 was selected [21], and it was enriched in 4 pathways as indicated in Fig. 5B. The binding sites between miR-26b-5p and TP53INP1 was verified by qRT-PCR (Fig. 5C). Cotransfection miR-26b-5p along with TP53INP1 3’-UTR wild-type significantly decreased luciferase activity of MG63/DDP and Saos2/DDP cells (Fig. 5D). TP53INP1 was enriched in miR-26b-5p biotin probe in contrast with negative control (Fig. 5E). Additionally, TP53INP1 expression were notably up-regulated by miR-26b-5p inhibitor while suppressed by miR-26b-5p mimic (Fig. 5F). TP53INP1 was suppressed in osteosarcoma cell lines compared to osteoblasts hFOB1.19. Furthermore, TP53INP1 expression level was further decreased in MG63/DDP and Saoa2/DDP cells (Fig. 5G).

### Inhibition of TP53INP1 alleviated the effects of down-regulated miR-26b-5p

**Fig. 6:**
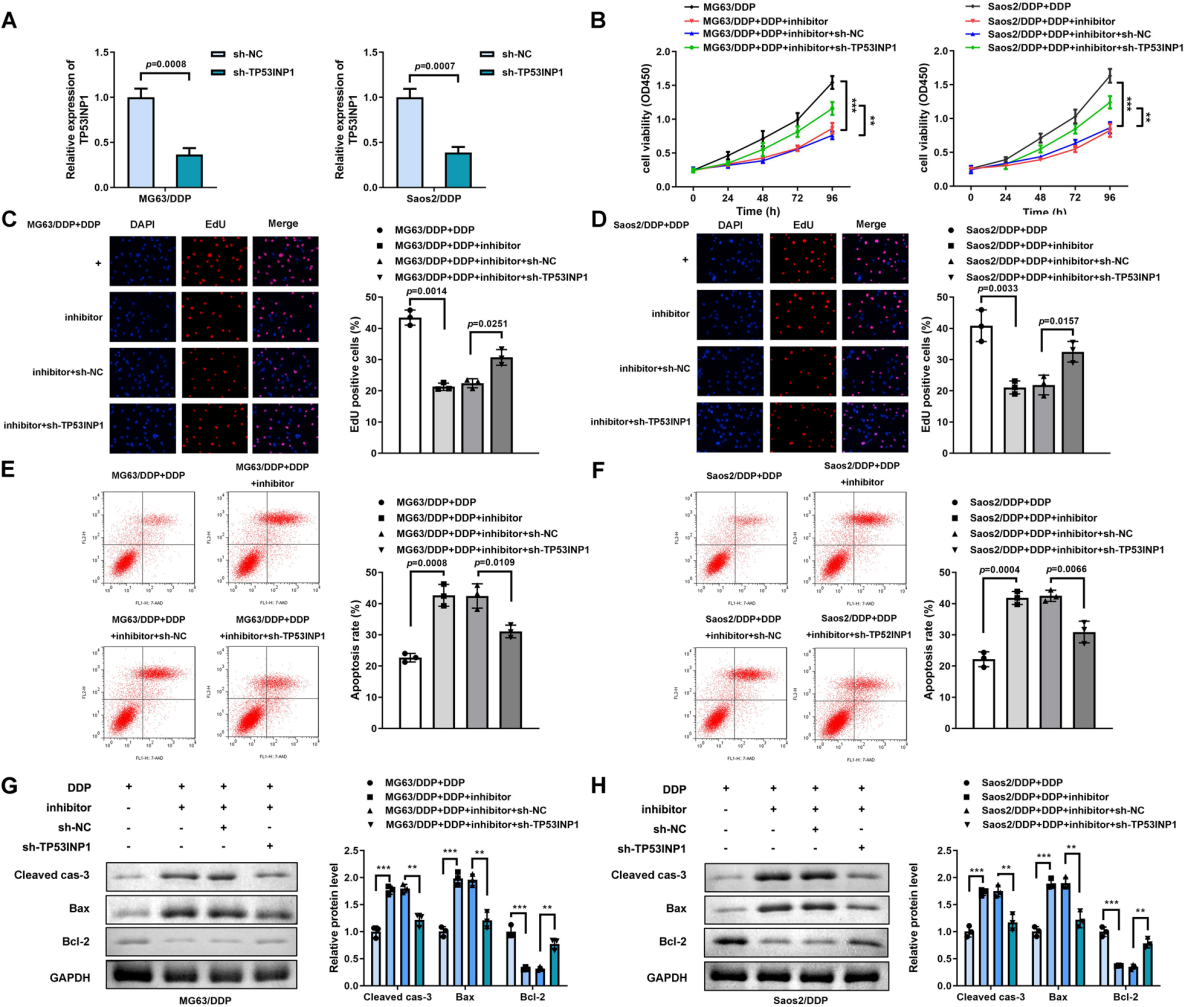
Inhibition of TP53INP1 alleviated the functions of down-regulated miR-26b-5p. **A** The expression levels of TP53INP1 of MG63/DDP and Saos2/DDP cells were detected by qRT-PCR after transfection. **B** Cell viability determined by CCK-8 assay in MG63/DDP and Saos2/DDP cells treated with DDP and co-transfected with sh-TP53INP1 and miR-26b-5p inhibitor, **P < 0.01; ***P < 0.001. **C**, **D**. Cell proliferation detected by EdU assay in MG63/DDP and Saos2/DDP cells treated with DDP and co-transfected with sh-TP53INP1 and miR-26b-5p inhibitor. **E**, **F** Cell apoptosis determined by flow cytometry assay in MG63/DDP and Saos2/DDP cells treated with DDP and co-transfected with sh-TP53INP1 and miR-26b-5p inhibitor. **G**, **H** Western blotting assay analyses of expression levels of caspase-3, Bax, as well as Bcl-2 in MG63/DDP and Saos2/DDP cells treated with DDP and co-transfected with sh-TP53INP1 and miR-26b-5p inhibitor, **P < 0.01; ***P < 0.001. The concentration of DDP is 2 μg/mL

The level of TP53INP1 was dramatically decreased in MG63/DDP and Saos2/DDP cells assessed by qRT-PCR after trasfection (Fig. 6A). Knockdown of TP53INP1 obviously abrogated the roles of miR-26b-5p inhibitor on cell proliferation (Fig. 6B–D) and cell apoptosis of MG63/DDP and Saoa2/DDP cells (Fig. 6E, F). Simultaneously, silenced TP53INP1 partly abrogated the regulatory function of suppressed miR-26b-5p on protein expression of apoptosis-related proteins (Fig. 6G, H).

## Discussion

In this study, GAS5 was found to suppress cell proliferation and accelerate cell apoptosis of DDP-resistant osteosarcoma cells by regulating miR-26b-5p/TP53INP1 axis. Furthermore, over-expression of GAS5 enhanced DDP sensitivity of osteosarcoma cells.

There is growing evidence that resistance to chemotherapy may be related to multiple factors. Downregulation of DNA-dependent protein kinase catalytic subunit can promote apoptosis of osteosarcoma cells and increase their sensitivity to cisplatin or etoposide [22]. Extracellular vesicles, especially exosomes participate in the proliferation and metastasis of osteosarcoma cells to regulate doxorubicin resistance [23–25]. Interestingly, circRNAs are also abundant and stably expressed in exosomes [25]. At present, the mechanisms of chemotherapy resistance of osteosarcoma remains unclear. Recently, large amount researched demonstrated that GAS5 is diffusely expressed in multitudinous tumor tissues, and is related to chemotherapy resistance. GAS5 can increase the resistance of mammalian targeted rapamycin (mTOR) antagonists in leukemia [26]. GAS5 sponges miR-21 to enhance the sensitivity of DDP treatment for cervical cancer [27]. In addition, GAS5 also plays an important role in regulating chemotherapy resistance in breast cancer, lung cancer, ovarian cancer, and other tumors [10, 11, 28, 29]. These findings suggested that GAS5 may be a chemotherapy sensitive gene. In this study, GAS5 was down-regulated in DDP-resistant tissues and DDP-resistant osteosarcoma cells, suggesting that down-regulated GAS5 may be related to DDP-resistant of osteosarcoma. Furthermore, GAS5 over-expression improved DDP therapeutic effects on DDP-resistant osteosarcoma cells, demonstrating that GAS5 enhanced DDP-sensitivity of osteosarcoma in vitro. In the study of melanoma, nanoparticles can deliver therapeutic agents specifically to the tumor microenvironment to play a therapeutic role [30]. The combination of GAS5 and nanotechnology to improve the chemotherapy sensitivity of osteosarcoma is also a topic worth exploring in future research.

Meanwhile, miRNAs play an important role in the pathogenesis of diverse human ailments [31, 32], and lncRNAs can serve as miRNA sponges and act as a new type of competing endogenous RNAs (ceRNA) regulator to degrade miRNA [33, 34]. Our data demonstrated that GAS5 could bind to miR-26b-5p, which has been investigated to serve as a regulator in various tumors. Khosla et al. [35] discovers that miR-26b-5p increases the malignancy of hepatocellular carcinoma by maintaining tumor stem cell characteristics. miR-26b-5p also suppresses the progression of human papillary thyroid cancer [36]. What’s more, Xie et al. [37] suggests that miR-26b-5p may take part in small cell osteosarcoma tumorigenesis. Additionally, miR-26b-5p enhances radiosensitivity of lung adenocarcinoma cells by suppressing transcription factor 2 protein [38]. Thence, the functions of miR-26b-5p is varied with tumor types. Nevertheless, whether miR-26b-5p could help to affect drug-resistance of oeteosarcoma remains to be investigated. In this study, GAS5 expression negatively regulated the miR-26b-5p expression. Moreover, over-expressed miR-26b-5p promoted the aggressiveness of DDP-resistant osteosarcoma cells. Hence, GAS5 enhanced the DDP sensitivity of osteosarcoma cells via miR-26b-5p.

TP53INP1 is an antioncogene that regulates the p53 response to stress [21]. Recent studies have found that TP53INP1 is also closely related to tumor drug resistance. TP53INP1 suppresses gemcitabine resistance in pancreatic cancer, sorafenib resistance and DDP resistance in liver cancer, paclitaxel resistance in breast cancer, DDP resistance in lung cancer, etc. [39–42]. Therefore, we speculated that TP53INP1 might be a new therapeutic target for chemotherapy resistance. In this study, TP53INP1 was found to be a downstream target gene of miR-26b-5p, and partly reversed the effects of miR-26b-5p on osteosarcoma cells. These results demonstrated that GAS5 sponged miR-26b-5p to up-regulate TP53INP1 to suppress DDP-resistance in osteosarcoma.

There are some limitations of our study. First of all, osterosarcoma tissues in this study were all from Han patients, so the sample capacity should be broadened and multiple ethnic groups needed to be supplied. The effect of GAS5 expression level in different stages of osteosarcoma can be elaborated. Secondly, in vivo experiment should be conducted in the future study to further investigate the role of elevated GAS5. Moreover, ferroptosis, necroptosis, and pyroptosis were also been observed in drug resistance of gastrointestinal cancers [43], which may also be investigated in DDP-resistance in osteosarcoma in the future.

## Conclusion

GAS5 was down-regulated and functioned as a cancer suppressor gene in osteosarcoma. Up-regulation of GAS5 contributed to the weakened malignancy in osteosarcoma cells and enhanced DDP chemoresistance by regulating miR-26b-5p/TP53INP1 axis. Up-regulation of GAS5 may be an effective therapeutic strategy for osteosarcoma.
